# Human and model observer performance for lesion detection in breast cone beam CT images with the FDK reconstruction

**DOI:** 10.1371/journal.pone.0194408

**Published:** 2018-03-15

**Authors:** Minah Han, Byeongjoon Kim, Jongduk Baek

**Affiliations:** School of Integrated Technology and Yonsei Institute of Convergence Technology, Yonsei University, Incheon, South Korea; University of Chicago Medical Center, UNITED STATES

## Abstract

We investigate the detectability of breast cone beam computed tomography images using human and model observers and the variations of exponent, *β*, of the inverse power-law spectrum for various reconstruction filters and interpolation methods in the Feldkamp-Davis-Kress (FDK) reconstruction. Using computer simulation, a breast volume with a 50% volume glandular fraction and a 2*mm* diameter lesion are generated and projection data are acquired. In the FDK reconstruction, projection data are apodized using one of three reconstruction filters; Hanning, Shepp-Logan, or Ram-Lak, and back-projection is performed with and without Fourier interpolation. We conduct signal-known-exactly and background-known-statistically detection tasks. Detectability is evaluated by human observers and their performance is compared with anthropomorphic model observers (a non-prewhitening observer with eye filter (NPWE) and a channelized Hotelling observer with either Gabor channels or dense difference-of-Gaussian channels). Our results show that the NPWE observer with a peak frequency of 7*cyc*/*degree* attains the best correlation with human observers for the various reconstruction filters and interpolation methods. We also discover that breast images with smaller *β* do not yield higher detectability in the presence of quantum noise.

## Introduction

Since breast cancer is the second leading cause of cancer-related deaths among women [1], early detection of breast cancer is very important. Mammography is most commonly used for breast cancer screening, but superimposing the breast anatomy structure onto its two-dimensional (2D) mammographic image reduces the accuracy of lesion detection [2, 3]. Breast cone beam computed tomography (CBCT) has been developed with the expectation that it will improve detection performance by reducing tissue superimposition in volumetric images [4–6].

Because of the potential of CBCT for breast imaging, evaluation of the detectability of breast CBCT images has become an important issue to optimize the imaging system. Past studies have investigated the effects of several imaging parameters on detectability, such as the X-ray tube voltage [7], scintillator thickness [8], detector pixel size [9], slice thickness [5, 10], and image plane [11]. Reconstruction algorithms producing optimal image quality in breast imaging have been explored [12–14], but the Feldkamp-Davis-Kress (FDK) algorithm [15] is the most widely used technique because of its simplicity, linearity, and computational efficiency. In the FDK reconstruction, optimal image quality can be acquired using various reconstruction filters and interpolation methods during back-projection depending on the task, and therefore, an investigation on the detectability for these parameters could help to optimize the FDK reconstruction in breast imaging.

In a previous study, it was reported that lesion detectability in breast CBCT images can be quantified by *β* (i.e. the slope of the breast anatomy power spectrum), because smaller *β* is considered as a predictor of better detectability [16–18]. A more desirable way to evaluate detectability in breast CBCT images is to conduct a human observer study because a human is the subject who makes diagnostic decisions. However, using human observers is time-consuming and expensive, thus it is desirable to use anthropomorphic model observers as surrogates [19–22]. In previous work [11], we evaluated detectability in breast CBCT images using *β* and model observers, especially for various reconstruction parameters considered in the FDK reconstruction, but without a human observer study.

In this study, we investigated the correlation between human observer and anthropomorphic model observer performance for lesion detection in breast CBCT images with various reconstruction filters and interpolation methods. The relationship between detectability by human observers and *β* was also investigated. For the investigations, we generated breast volumes with 50% volume glandular fraction (VGF) and a 2*mm* diameter lesion using computer simulation. Three reconstruction filters: Hanning, Shepp-Logan, and Ram-Lak [23] were considered in the FDK reconstruction, and projection data were filtered with and without Fourier interpolation before back-projection. We conducted signal-known-exactly and background-known-statistically (SKE/BKS) detection tasks. To evaluate detectability, we performed human observer experiments with four-alternative forced choice (4AFC) detection tasks [24]. For the model observer study, we used a non-prewhitening observer with eye filter (NPWE) [25] and a channelized Hotelling observer (CHO) with either Gabor channels (Gabor CHO) [26] or dense difference-of-Gaussian channels (D-DOG CHO) [27]. To present the Hotelling observer performance, we used Laguerre-Gauss channels (LG CHO) [28].

## Methods

### Image generation

#### Simulated breast volume

Breast volumes were generated in computer simulations using two main characteristics of breast anatomy: 1) a power law spectrum and 2) binary attenuation coefficients. The structure of the breast anatomy in mammograms has been characterized using the power law spectrum [16, 18]

$$\begin{array}{c} P\left(f\right)=K/f^{\beta } \end{array}$$ (1)

where *f* is the radial frequency (*mm*^−1^), *K* is a constant and *β* is a power law exponent. Based on real clinical mammograms, the estimated value of *β* is ∼3 [16]. The attenuation coefficient of breast anatomy is considered as binary to represent the dominant glandular and adipose tissues [29].

For the breast volume, we generated a volume with 512×512×512 voxels of white Gaussian noise and transformed it into the frequency domain using the discrete Fourier transform (DFT). We computed a pointwise multiplication of the transformed white noise and a three-dimensional (3D) filtering kernel (i.e. the square root of 1/*f*^3^), and then calculated the inverse DFT. Note that Eq (1) is a 2D power spectrum, and we simply extended it to the 3D power spectrum [6, 11] by the central slice theorem [30, 31]. As *f* approaches 0, the value of the filtering kernel becomes infinite; thus, the value at *f* = 0 was set to twice that of the first nonzero radial frequency component [17]. We extracted the central spherical volume with a diameter of 128 voxels from the filtered noise to avoid the wrap-around effect owing to the DFT filtering operation [27]. Afterwards, we sorted the voxel values of the spherical volume in descending order, and assigned the attenuation coefficient of glandular tissue to the top 50% of voxel values and that of adipose tissue to the remaining 50% [32–34], representing a 50% VGF breast volume.

For modelling a mass lesion, a 2*mm* diameter spherical signal was inserted near the center of the breast volume by replacing the attenuation coefficient of the breast tissue in the signal region with that of the signal. The attenuation coefficients of simulated glandular and adipose tissues, and signal region were 0.233, 0.194, and 0.238*cm*^−1^, respectively, which were attenuation coefficients at 50keV energy [35]. The image voxel size was 0.16×0.16×0.16*mm*^3^ and the full volume size was 20.5×20.5×20.5*mm*^3^.

#### CBCT data acquisition

We computed the radiological path along the ray that connected the X-ray source and each of the detector pixels to acquire projection data of the simulated breast volume [36] (the CBCT simulation parameters are summarized in Table 1), and a detector quarter offset was used to avoid aliasing. For quantum noise, we generated uniform Poisson noise with 6914 photons per detector pixel, equivalent to the dose used in two-view mammography (i.e. 6.4*mGy* for a 14*cm* diameter breast with 50% VGF) [37]. Next, we applied log-normalization to the generated Poisson noise and added it to the noiseless projection data.

**Table 1:** CBCT simulation parameters.

Parameter	Value
Source to Iso-center Distance	460*mm*
Source to Detector Distance	880*mm*
Detector pixel size	0.388×0.388*mm*^2^
Detector array size	150×150 pixels (i.e., 58.2×58.2*mm*^2^)
Number of Views	200
Reconstructed voxel size	0.1014×0.1014×0.1014*mm*^3^
Reconstructed matrix size	200×200×200 voxels
Reconstructed volume size	20.3×20.3×20.3*mm*^3^

In a discrete-to-discrete projection procedure, discretization artifacts in the projection of the breast volume can be introduced when the image voxel size is larger than the detector pixel size [32]. To avoid these, the voxel size of the simulated breast volume was set to 0.16×0.16×0.16*mm*^3^, which was smaller than the detector pixel size magnified at the iso-center (0.2028×0.2028*mm*^2^) [11, 32].

**Fig 1:**
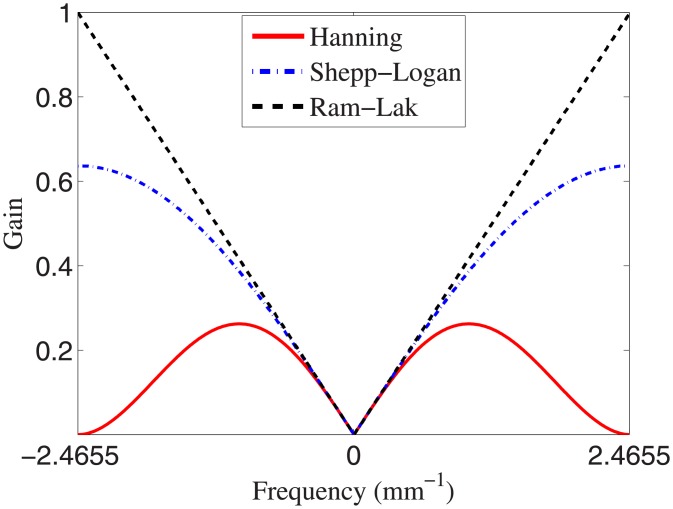
Profiles of the reconstruction filters.

Projection data were reconstructed using the FDK reconstruction [15] in which the voxel size was selected as half of the intrinsic voxel size at the iso-center. In the FDK reconstruction, the ramp filter was apodized using three filters: Hanning, Shepp-Logan, or Ram-Lak [23]; their frequency profiles are shown in Fig 1. To examine the effect of linear interpolation on detectability [38], projection data were filtered with and without 8-fold Fourier interpolation, and then voxel-driven back-projection with linear interpolation was performed. Note that the 8-fold Fourier interpolation was implemented by taking the inverse Fourier transform of the zero padded (seven times the length of the filtered projection data length) Fourier transform of the filtered projection data.

#### Image preparation

**Fig 2:**
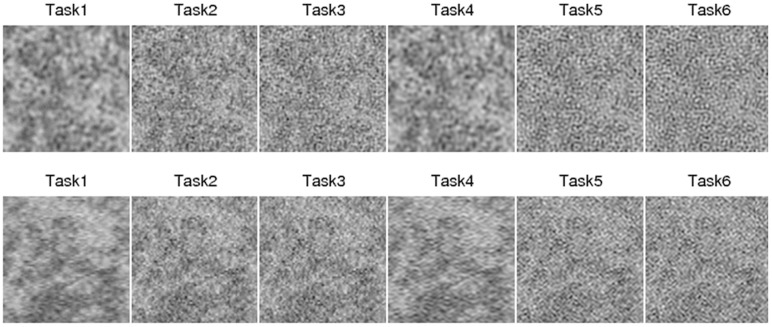
Reconstructed CBCT images. Reconstructed CBCT images for the transverse (upper) and the longitudinal plane (lower). The display window was [0.10, 0.39] *cm*^−1^.

To evaluate detectability, we extracted a central volume of 128×128×128 voxels from the full volume of 200×200×200 voxels. Next, we used the central transverse (x-y) plane and the longitudinal (x-z) plane of the volume. With three reconstruction filters and two interpolation methods, evaluation of a total of six detection tasks was conducted for the transverse and longitudinal planes, as summarized in Table 2. The reconstructed breast images for the six tasks are shown in Fig 2.

**Table 2:** Reconstructed image sets for detection task with different reconstruction filters and interpolation methods.

Task	Reconstruction filter	Interpolation method
1	Hanning	Linear
2	Shepp-Logan	Linear
3	Ram-Lak	Linear
4	Hanning	Fourier
5	Shepp-Logan	Fourier
6	Ram-Lak	Fourier

### 4AFC detection task

To evaluate detectability by the human and model observers, we conducted 4AFC SKE/BKS detection tasks. The two hypotheses (i.e. *H*_0_ and *H*_1_ for signal-absent and signal-present, respectively) are given by [39]:

$$\begin{array}{c} H_{0}:g=f_{b}+f_{n} \end{array}$$ (2)

$$\begin{array}{c} H_{1}:g=f_{s}+f_{n} \end{array}$$ (3)

where **f**_**b**_ is the breast background, **f**_**s**_ is the breast background containing the 2*mm* signal, **f**_**n**_ is the noise in the reconstructed CT image, and **g** is the transverse or longitudinal plane reconstructed with various reconstruction filters and interpolation methods.

### The human observer study

**Fig 3:**
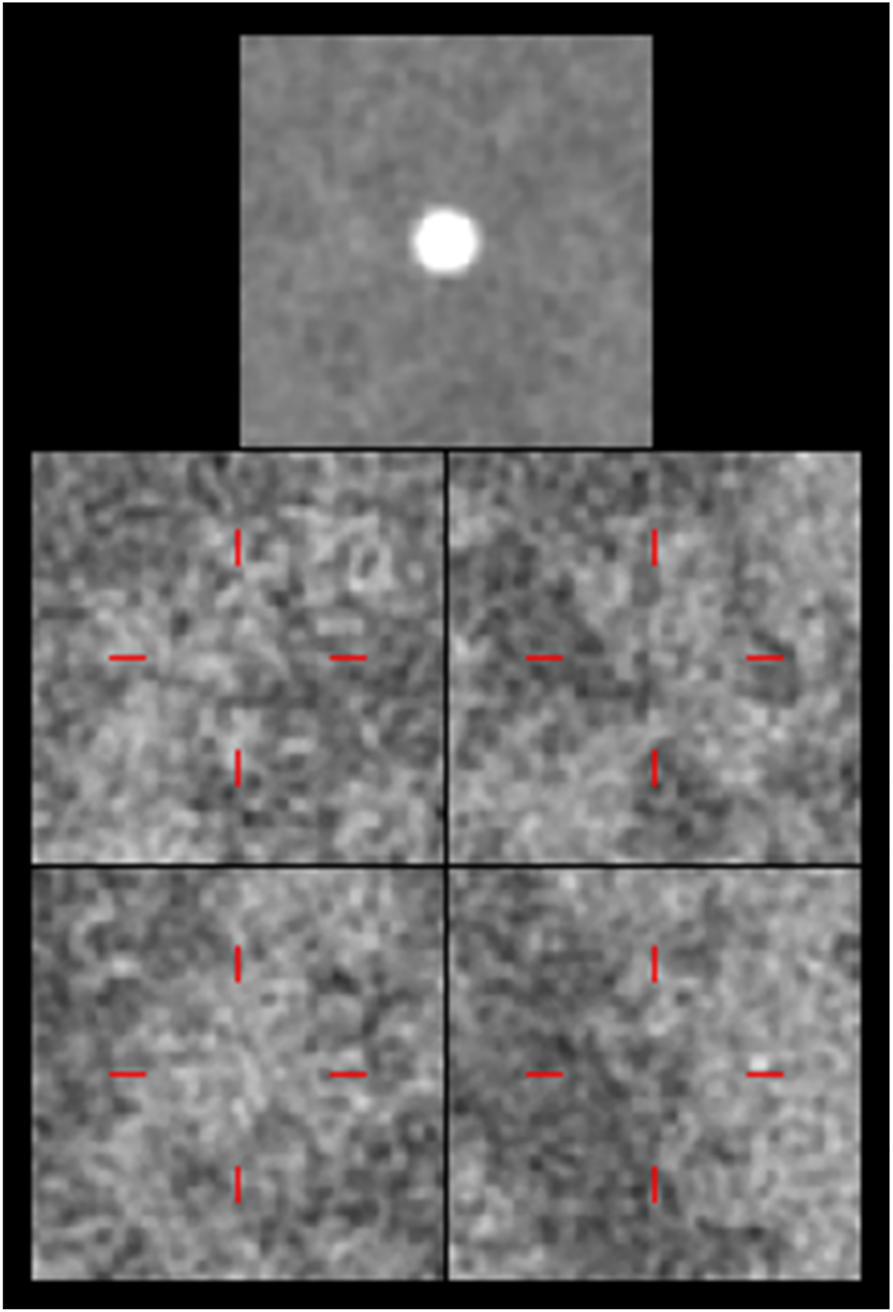
The 4AFC detection task for the human observer study.

For the human observer study, seven human observers participated and performed six detection tasks for the transverse and longitudinal planes. In each trial, the observers were shown a signal image [20, 40], one signal-present image and three signal-absent images, as shown in Fig 3, and then asked to select the signal-present image. Images were displayed on a 21.3 inch Nio 3MP LED monitor (Barco, Kortrijk, Belgium) with a resolution of 2048×1536 pixels. To reduce distraction, the area around the test images was filled with a dark background. The locations of the signal-present image and signal-absent images were randomly switched in each trial. For all images, the area where the signal may have been present (i.e. the center of the image) was indicated with a red cross. For each task, the observers were trained with 30 trials with instant feedback and tested with 100 trials. We used unique 100 signal-present and 300 signal-absent image datasets for each task and the images were randomly selected from 400 image volumes and displayed in a random order. Note that the training dataset was generated independently from the test dataset for each task, and the training and test datasets were independent for each observer. No time limit was set for choosing an image. Although there was no restriction on viewing distance, it remained approximately 40∼50*cm*.

For the *j* − *th* trial of each observer, score *o*_*j*_ was recorded, where *j* = 1, 2, …, 100. If the answer was correct, *o*_*j*_ was 1, else it was 0. Subsequently, the percent correct, *P*_*c*_, was computed by

$$\begin{array}{c} P_{c}=\frac{1}{N_{t}}\sum_{j=1}^{N_{t}}o_{j} \end{array}$$ (4)

where *N*_*t*_ is the number of test trials, and the variance of *P*_*c*_ was estimated by bootstrapping the scores 1,000 times [24].

### Model observer study

To compare the detectability by the human observer with that of the model observer, we considered three anthropomorphic model observers: NPWE [25], Gabor CHO [26] and D-DOG CHO [27] because the eye filter in NPWE, and the Gabor and D-DOG channels in CHO mimic the human visual system [19, 40]. Since our signal was rotational symmetric and the background was stationary within the field of view, we used LG CHO to represent the detectability of the Hotelling observer [19, 20].

#### The template for NPWE

NPWE applies a band pass filter (i.e. eye filter) to images which mimic the frequency selective characteristics of the human visual system [25]. The eye filter function is defined as [25, 27]

$$\begin{array}{c} E\left(f\right)=f^{1.3}exp\left(-cf^{2}\right) \end{array}$$ (5)

where *c* is an eye filter parameter.

The template of NPWE is given by

$$\begin{array}{c} w_{\text{NPWE}}=F^{-1}\{E\cdot F\{\Delta g\}\} \end{array}$$ (6)

where *F*{⋅} is a DFT operator, *F*^−1^{⋅} is an inverse DFT operator, and **Δg** is the mean difference between signal-present and signal-absent images. To estimate **Δg**, we used 400 signal-present and signal-absent image pairs.

#### The template for CHO

CHO applies multiple channels to images and generates a channelized image given by

$$\begin{array}{c} v=Tg \end{array}$$ (7)

where **v** is the channelized image, **T** is the channel matrix and **g** is the given image.

For Gabor CHO, the Gabor function is defined as

$$\begin{array}{c} Ch\left(x,y\right)=exp\left[-4\left(ln2\right)\left({\left(x-x_{0}\right)}^{2}+{\left(y-y_{0}\right)}^{2}\right)/w_{s}^{2}\right]\times cos\left[2\pi f_{c}\left(\left(x-x_{0}\right)cos\theta +\left(y-y_{0}\right)sin\theta \right)+\xi \right] \end{array}$$ (8)

where (*x*_0_, *y*_0_) is the center of the channel and is set to the center of the signal, *w*_*s*_ is the spatial width of the channel, *f*_*c*_ is the center frequency of the channel, *θ* is the channel orientation, and *ξ* is a phase factor [26]. Channel matrix **T** of the Gabor CHO is composed of discrete samples obtained from Eq (8).

For D-DOG CHO, the DOG function is in the form

$$\begin{array}{c} C_{j}\left(\rho \right)=exp\left[-\frac{1}{2}{\left(\frac{\rho }{Q\sigma_{j}}\right)}^{2}\right]-exp\left[-\frac{1}{2}{\left(\frac{\rho }{\sigma_{j}}\right)}^{2}\right] \end{array}$$ (9)

where *ρ* is the radial frequency (pixel^−1^), *Q* is a multiplicative factor, and *σ*_*j*_ is the standard deviation of the *j* − *th* channel defined as *σ*_*j*_ = *σ*_0_ *α*^*j*^. Channel matrix **T** of the D-DOG CHO is composed of discrete samples of the inverse DFT of Eq (9).

For LG CHO, the LG function is defined as

$$\begin{array}{c} u_{p}\left(r|a_{u}\right)=\frac{\sqrt{2}}{a_{u}}exp\left(\frac{-\pi r^{2}}{a_{u}^{2}}\right)L_{p}\left(\frac{2\pi r^{2}}{a_{u}^{2}}\right) \end{array}$$ (10)

where **r** represents a 2D spatial coordinate and *a*_*u*_ is the width of the Gaussian function. The Laguerre polynomials, *L*_*p*_(*x*), can be defined as

$$\begin{array}{c} L_{p}\left(x\right)=\sum_{k=0}^{p}{\left(-1\right)}^{k}\left(\begin{array}{l} p\\ k \end{array}\right)\frac{x^{k}}{k!} \end{array}$$ (11)

where *p* is the order of the polynomial. Channel matrix **T** of the LG CHO is composed of discrete samples obtained from Eq (10).

The template of the CHO is given by

$$\begin{array}{c} w_{\text{CHO}}={\left(K_{v}+K_{\text{int}}\right)}^{-1}\Delta v \end{array}$$ (12)

where **K**_**v**_ is the channelized covariance matrix, **Δv** is the channelized mean difference between the signal-present and signal-absent images, and **K**_**int**_ is the covariance matrix of channel internal noise given as

$$\begin{array}{c} K_{\text{int}}=a\cdot (I\;{}^{\circ}\;K_{v}) \end{array}$$ (13)

where ° is an elementwise multiplication operator, *a* is the internal noise level, and **I** is an identity matrix. We used a nonuniform channel internal noise model since it showed good agreement between the human and model observers [41]. To estimate **K**_**v**_ and **Δv**, we used 400 signal-present and signal-absent image pairs. Note that the image sets used to estimate **K**_**v**_ and **Δv** were independent of each other.

#### *P*_*c*_ of the model observers

Model observer performance was evaluated with the 4AFC task. Each trial was composed of one signal-present and three signal-absent images. A test consisting of 100 trials was performed independently seven times. For each trial, the decision variable for NPWE is computed by

$$\begin{array}{c} t={w_{\text{NPWE}}}^{t}g_{\text{eye}}+\varepsilon \end{array}$$ (14)

where **g**_**eye**_ = *F*^-1^{**E** · *F*{**g**}} and *ε* is the internal noise.

The decision variable for the CHO is derived as

$$\begin{array}{c} t={w_{\text{CHO}}}^{t}\left(v+v_{\text{int}}\right) \end{array}$$ (15)

where **v**_**int**_ is the channel internal noise. Note that the *j* − *th* element of **v**_**int**_ was sampled from random noise whose variance is equivalent to the (*j*, *j*) element of $\sqrt{K_{\text{int}}}$.

For each trial, the image which produced the highest decision variable among the four images was determined as a signal-present image. If the answer from the model observer was correct, then *o*_*j*_ = 1, else *o*_*j*_ = 0. *P*_*c*_ was computed using Eq (4), and its variance was estimated by bootstrapping the scores 1,000 times. With internal noise (*ε*>0 and *a*>0), *P*_*c*_ was computed by averaging 10 repetitions of the *P*_*c*_ calculation.

#### Parameter selection for the model observers

The model observer have several parameters but adjusting all of them based on human observer data increases complexity and produces overfitting problems; thus, we adjusted only one parameter for each model observer.

For NPWE, we used two models. In the first model, denoted as NPWE4i, we adjusted the internal noise level *ε* with *c* = 2 such that the peak value of the eye filter occurred at 4*cyc*/*degree* because this is the value at which the human visual system is most sensitive [27]. In the second model, denoted as NPWEf, we adjusted the peak frequency of the eye filter by changing the *c* value and set *ε* = 0.

For Gabor CHO, we adjusted internal noise level *a* with *f*_*c*_ = 3/128, 3/64, 3/32 and 3/16 (*w*_*s*_ = 56.48, 28.24, 14.12 and 7.06), *θ* = 0, 2*π*/5, 4*π*/5, 6*π*/5 and 8*π*/5, and *ξ* = 0 and *π*/2, as used in [26].

For D-DOG CHO, we adjusted *a* with *σ*_0_ = 0.005, *α* = 1.4, *Q* = 1.67, and *j* = 1∼10, as used in [27].

Optimal values of *ε*, *c*, and *a* were selected to minimize the mean squared error (MSE) of *P*_*c*_ between the model and human observers. The searching interval for *ε* was 0.001 within a range of [0, 0.003], and the searching interval for *c* and *a* was 0.1 within a range of [0, 3] and [0, 5], respectively.

For the LG CHO, we used 10 channels with *a*_*u*_ = 6 and *a* = 0, because *P*_*c*_ became saturated when the number of channels was greater than 10, and was maximized with *a*_*u*_ = 6. The description of the model observers used in this study are summarized in Table 3.

**Table 3:** The model observers used in this study.

Model observer	Description
NPWE4i	NPWE with a peak frequency at 4*cyc*/*degree* and internal noise
NPWEf	NPWE without internal noise and adjusted peak frequency
Gabor CHOi	Gabor CHO with internal noise
D-DOG CHOi	D-DOG CHO with internal noise
LG CHO	LG CHO without internal noise

### Estimation of *β*

To estimate *β*, we first computed a 2D noise power spectrum (NPS) using 500 reconstructed signal-absent breast images. For each image, we subtracted the mean and multiplied the image by a spatial window **W** to avoid spectral leakage [18] owing to the finite length of the DFT.

$$\begin{array}{c} W\left(r\right)=\{\begin{array}{cc} 0.5+0.5cos(\pi r/D) & r\leq D\\ 0 & r>D \end{array} \end{array}$$ (16)

Next, we performed the DFT on the window-applied images, and computed the 2D NPS by averaging the squared magnitudes of the DFT images. We computed a 1D NPS by radial averaging of the 2D NPS, and then applied the natural logarithm to the 1D NPS. To obtain *β*, we performed linear regression on the logarithm-applied 1D NPS by changing the fitting frequency range. The value of *β* was chosen to maximize the coefficient of determination (*R*^2^) [18]. Note that *R*^2^ measures the correspondence between a logarithm-applied 1D NPS and the corresponding linear regression model.

## Results

**Fig 4:**
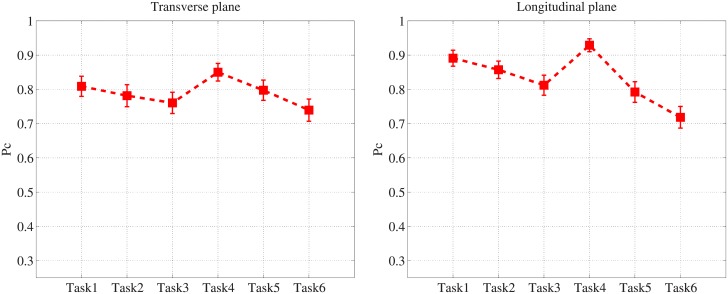
*P*_*c*_ by human observer. Averaged *P*_*c*_ values of human observers with 95% confidence intervals.

**Fig 5:**
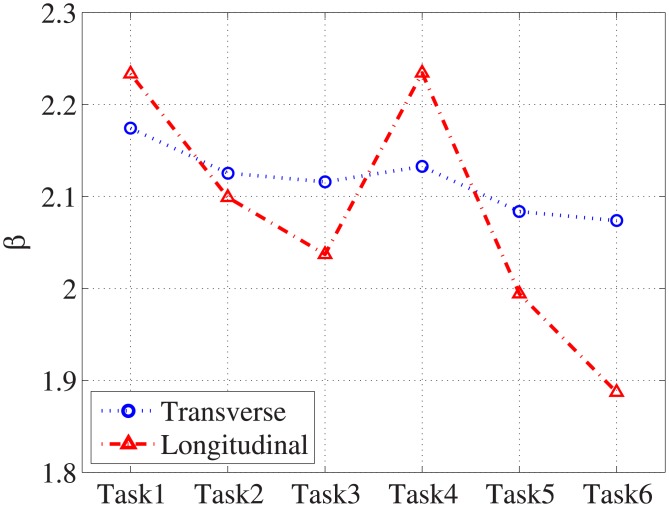
Estimated *β*. *β* of the transverse and longitudinal planes.

**Fig 6:**
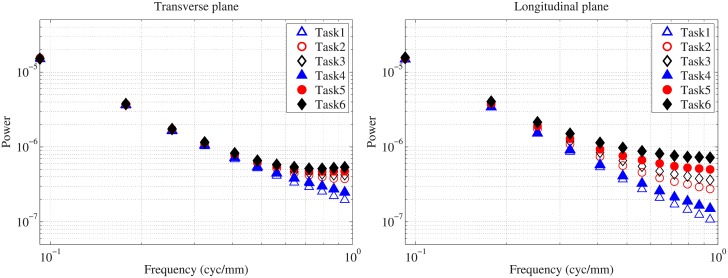
1D NPS. Log-log plots of 1D NPS for the transverse and longitudinal planes.

Fig 4 shows the averaged *P*_*c*_ of the human observers and Fig 5 shows the measured *β* for each task. For *β* estimation, the fitting frequencies ranged from 0.1 to 0.48*cyc*/*mm* with *R*^2^>0.95. For the various reconstruction filters, the averaged *P*_*c*_ is highest (lowest) when the Hanning (Ram-Lak) filter is used because the Hanning (Ram-Lak) filter yields the lowest (highest) noise power, as shown in Fig 6. Note that the 1D NPS shown in Fig 6 is up to 1*cyc*/*mm* because more than 99% signal power is concentrated below 1*cyc*/*mm*. While the image with the Hanning filter produces the highest *P*_*c*_, *β* is also at its highest for this image. Since the Hanning filter reduces high-frequency energy more than the other filters, the slope of the corresponding logarithm-applied 1D NPS increases, which yields a higher *β*.

The interpolation method producing higher *P*_*c*_ depends on reconstruction filters and image planes, while the Fourier interpolation reduces *β* for all reconstruction filters and image planes. The frequency response of linear interpolation (*sinc*^2^(*f*)) reduces high-frequency energy, resulting in a higher *β*, but using Fourier interpolation minimizes the effect of linear interpolation.

For the image planes, the longitudinal plane produces higher *P*_*c*_ than the transverse plane from tasks 1 to 4, and the transverse plane produces higher *P*_*c*_ for tasks 5 and 6, because the noise power of the longitudinal plane is higher than that of the transverse plane for these two tasks. The variation of *P*_*c*_ for the various tasks is larger in the longitudinal plane than in the transverse plane because the noise power varies more significantly in the longitudinal plane, as shown in Fig 6. As a result, the *β* value varies more in the longitudinal plane for the various tasks, as shown in Fig 5.

**Fig 7:**
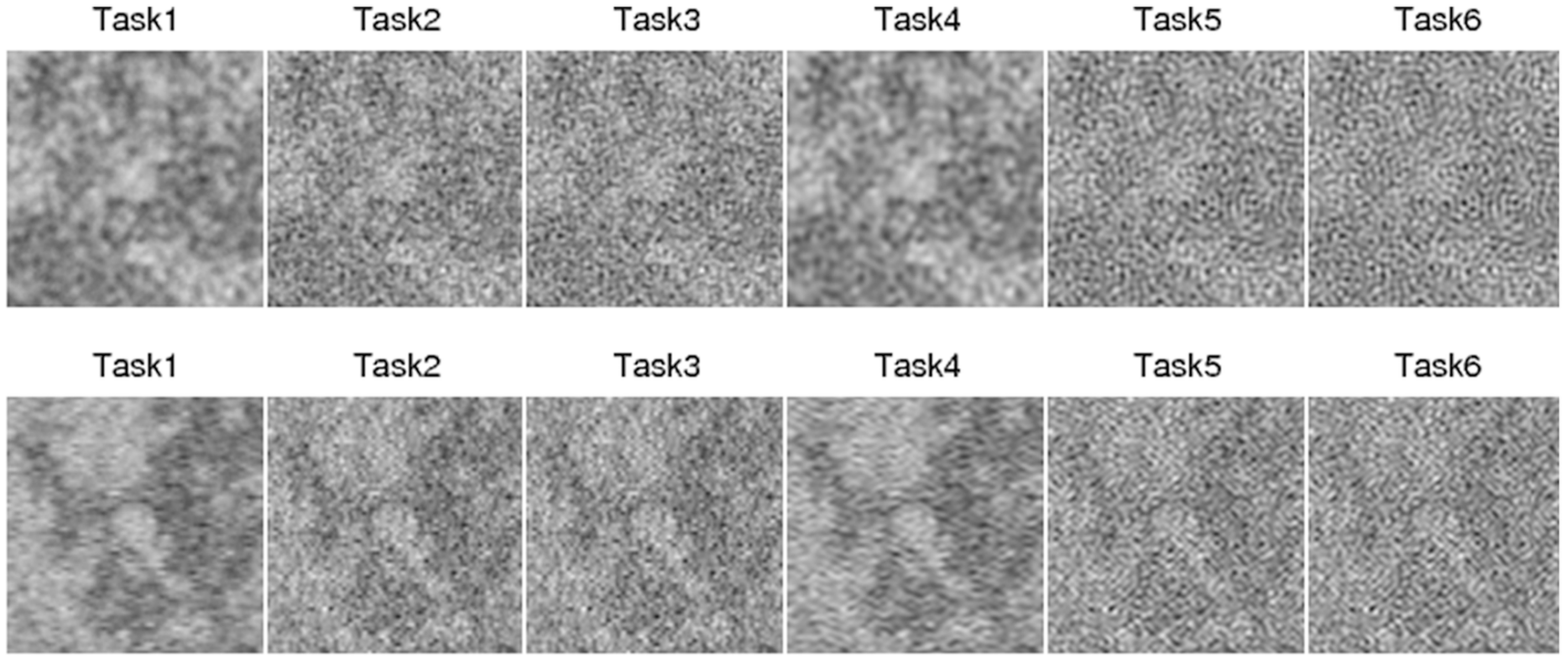
Example CBCT images. Example images with a 2*mm* diameter signal for the transverse (upper) and longitudinal planes (lower).

For both image planes, *P*_*c*_ is highest in task4 and lowest in task6, and Table 4 contains a summary of *P*_*c*_ and *β* for these tasks. This shows that a task with higher *P*_*c*_ has higher *β*, which is different from the conclusion of the previous study (i.e., higher *β* implies lower detectability [16, 17]). This contradiction mainly comes from using apodization filters in the FDK algorithm and the presence of quantum noise, which have not been considered in traditional mammography studies. Quantum noise is a limiting factor in CT image quality because the quantum noise level of each CT projection is much higher than that of the mammography with an equivalent dose assumption. For a qualitative comparison, Fig 7 shows example reconstructed images with the signal for the various tasks.

**Table 4:** *P*_*c*_ and *β* of the task which produces the highest *P*_*c*_ (task4) and lowest *P*_*c*_ (task6).

	Transverse		Longitudinal	
	*P*_*c*_	*β*	*P*_*c*_	*β*
Task4	0.85	2.13	0.93	2.23
Task6	0.74	2.07	0.72	1.89

**Fig 8:**
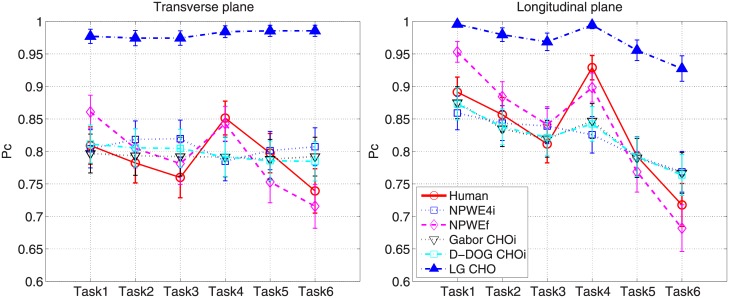
*P*_*c*_ by human and model observers. The *P*_*c*_ value of the human and model observers with 95% confidence intervals.

**Fig 9:**
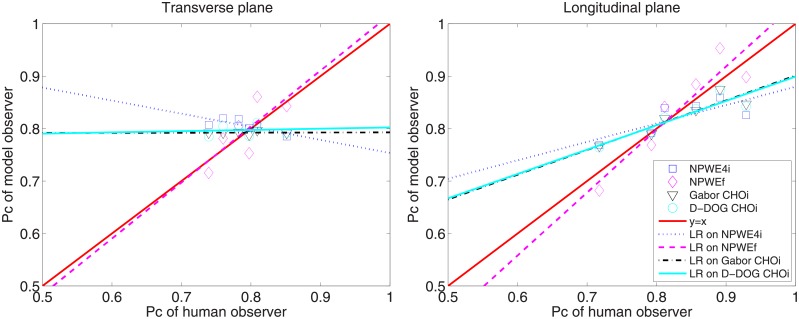
Correlation between human and model observers. Scatter plots of *P*_*c*_ between human and model observers, and corresponding linear regression (LR) results.

**Fig 10:**
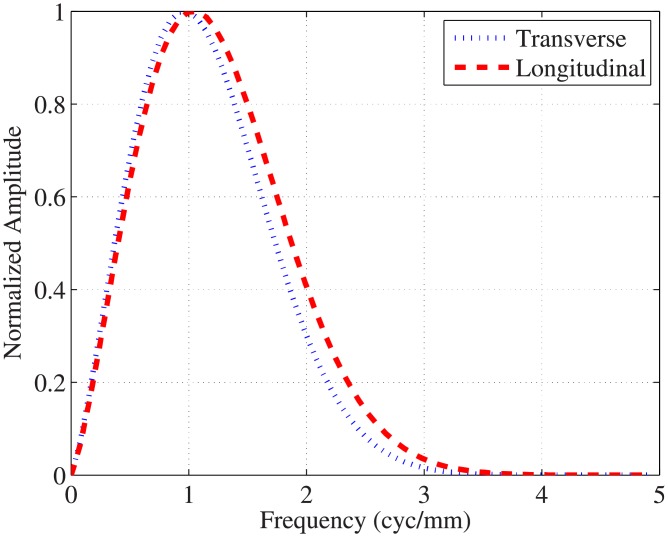
Eye filter. Eye filter profiles with the optimal value of *c*.

Fig 8 shows the *P*_*c*_ values for the human and model observers. Note that *P*_*c*_ for the anthropomorphic model observers (NPWE4i, NPWEf, Gabor CHOi and D-DOG CHOi) in Fig 8 is calculated using the optimal *ε*, *c*, and *a* values summarized in Table 5. LG CHO shows the highest *P*_*c*_ value for all tasks and image planes. Fig 9 shows scatter plots of *P*_*c*_ between the human and anthropomorphic model observers, and corresponding linear regression lines. The greater the similarity between the linear regression line and *y* = *x* line, the higher the correlation between the human and model observers. NPWEf shows the best correlation with a human observer in both the transverse and longitudinal planes; the eye filter profiles for NPWEf with optimal *c* are shown in Fig 10. The eye filter has its peak value at 1*cyc*/*mm*, which translates to 7*cyc*/*degree* for a 400*mm* viewing distance belonging to the sensitive frequency range of the human visual system [42].

**Table 5:** Optimal parameters (i.e., *ε*, *c* and *a*) for the anthropomorphic model observers.

	NPWE4i	NPWEf	Gabor CHOi	D-DOG CHOi
Transverse	*ε* = 0.001	*c* = 0.7	*a* = 2.9	*a* = 3.8
Longitudinal	*ε* = 0.001	*c* = 0.6	*a* = 2.1	*a* = 2.6

## Conclusion and discussion

We evaluated detectability in breast CBCT images using human and model observers, and *β* with various reconstruction filters, interpolation methods, and image planes. In this study, NPWEf with a peak frequency of 7*cyc*/*degree* showed the highest correlation with human observer performance for the various reconstruction filters, interpolation methods, and image planes. For both image planes, detectability was highest for the Hanning filter and lowest for the Ram-Lak filter both with Fourier interpolation. However, higher detectability was not related to a smaller *β* value in the presence of quantum noise.

**Fig 11:**
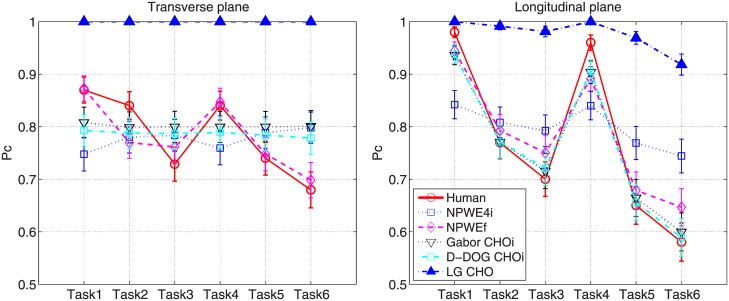
*P*_*c*_ by human and model observers for uniform background. *P*_*c*_ of human and model observers with 95% confidence intervals for uniform backgrounds.

Although the main focus of this work is to evaluate the detectability in breast CBCT images, we evaluated detectability for the same tasks with a uniform background. Note that the 2*mm* diameter spherical signal was also used for the uniform background case, and the intensity of the signal was adjusted to produce *P*_*c*_ values ranging from 0.5 to 1 for all tasks. As shown in Fig 11, the NPWEf still showed a high correlation with the human observers for both image planes. Note that the optimal eye filter for a uniform background had a peak value at around 1.5*cyc*/*mm*, which is different from the breast anatomy background (1*cyc*/*mm* as shown in Fig 10). This result indicates that the peak frequency of an eye filter depends on image statistics. In contrast to the results with breast CBCT images, both Gabor CHOi and D-DOG CHOi showed good correlation with human observers for the longitudinal plane.

Of the model observers, NPWEf showed the highest correlation with human observers, but its performance was sensitive to the peak frequency of the eye filter. Thus, optimization for one signal size might not have been optimal for other signal sizes. In these cases, D-DOG CHOi would be more useful to predict human observer performance, as shown in [20].

We used a central single slice of the CBCT images, but in order to take signal information along the direction in which the image slices are stacked into account, evaluating multiple slices of CBCT images is required, which is a subject for future research.

Since our study is a preliminary investigation of detectability in breast CBCT images, we modelled the breast volume using simple characteristics of breast anatomy without considering detailed morphological features such as tissue orientation [43], other tissue types [44], various VGF values [45], and other shapes of lesion [46]. In our CBCT simulation, we did not consider the effect of beam hardening and scatter because their effects on image quality are dependent on X-ray energy, object size, and data acquisition geometry. In the presence of beam hardening and scatter, detectability will be reduced due to the decreased contrast, especially for low contrast small objects. However, the effects of the apodization filter and interpolation method on detection task would remain the same. We also assumed an ideal detector response. Correlation between detector pixels and nonuniform detector pixel response degrade the overall image quality, and thus provide lower detectability in our tasks. In future studies, we aim to extend this work to clinical breast CBCT images, for which we will consider these physical factors.

## Supporting information

S1 File *P*_*c*_ by each of the seven human observers. *P*_*c*_ values by each of the seven human observers for six detection tasks.
(XLSX)
